# Dispersion of single-wall carbon nanotubes with supramolecular Congo red – properties of the complexes and mechanism of the interaction

**DOI:** 10.3762/bjnano.8.68

**Published:** 2017-03-16

**Authors:** Anna Jagusiak, Barbara Piekarska, Tomasz Pańczyk, Małgorzata Jemioła-Rzemińska, Elżbieta Bielańska, Barbara Stopa, Grzegorz Zemanek, Janina Rybarska, Irena Roterman, Leszek Konieczny

**Affiliations:** 1 Chair of Medical Biochemistry, Faculty of Medicine, Jagiellonian University Medical College, Kopernika 7, Kraków 31-034, Poland; 2 Institute of Catalysis and Surface Chemistry, Polish Academy of Science, Niezapominajek 8, Kraków 30-239, Poland; 3 Department of Plant Physiology and Biochemistry, Faculty of Biochemistry, Biophysics and Biotechnology, Jagiellonian University, Krakow, Poland; 4 Malopolska Centre of Biotechnology, Jagiellonian University, Krakow, Poland; 5 Department of Bioinformatics and Telemedicine, Jagiellonian University Medical College, św. Łazarza 16, Kraków 31-034, Poland

**Keywords:** Congo red, single-wall carbon nanotubes, supramolecular compounds

## Abstract

A method of dispersion of single-wall carbon nanotubes (SWNTs) in aqueous media using Congo red (CR) is proposed. Nanotubes covered with CR constitute the high capacity system that provides the possibility of binding and targeted delivery of different drugs, which can intercalate into the supramolecular, ribbon-like CR structure. The study revealed the presence of strong interactions between CR and the surface of SWNTs. The aim of the study was to explain the mechanism of this interaction. The interaction of CR and carbon nanotubes was studied using spectral analysis of the SWNT–CR complex, dynamic light scattering (DLS), differential scanning calorimetry (DSC) and microscopic methods: atomic force microscopy (AFM), transmission (TEM), scanning (SEM) and optical microscopy. The results indicate that the binding of supramolecular CR structures to the surface of the nanotubes is based on the "face to face stacking". CR molecules attached directly to the surface of the nanotubes can bind further, parallel-oriented molecules and form supramolecular and protruding structures. This explains the high CR binding capacity of carbon nanotubes. The presented system – containing SWNTs covered with CR – offers a wide range of biomedical applications.

## Introduction

Carbon nanotubes (CNTs) present enormous application potential in many areas of chemistry, technology and medicine and are currently one of the most intensely studied nanomaterials. Biomedical use of CNTs includes biosensors [1], bioimaging [2–3], drug delivery [4–9] and tissue engineering [10–12]. Pristine CNTs exist in form of bundles composed of hundreds of single tubes bound by van der Waals interactions. Most applications of carbon nanotubes require their dispersion (solubilization) which can be achieved by either covalent or noncovalent functionalization [8,13–14]. Covalent functionalization modifies CNT walls through introduction of different atoms or groups (e.g., fluoride, carboxyl, hydroxyl) to the carbon lattice. Noncovalent functionalization leaves the nanotube walls intact, but allows for separation of the bundles due to their interaction with different amphiphilic molecules that cover the hydrophobic CNT surface with hydrophilic groups [15].

Functionalization leads not only to the increased water dispersibility of CNTs but also improves their biocompatibility due to enhanced penetration through biological membranes and reduced cytotoxicity [16–17]. Functionalization also allows for the attachment of biologically active molecules – e.g., drugs, nucleic acids, antibodies or ligands for cell-surface receptors. This is especially important for targeted drug delivery systems based on CNT [13,15,18–21]. Noncovalent functionalization of CNTs is usually performed by sonication of pristine CNTs in an amphiphilic solution. Sonication leads both to the solubilization and shortening of carbon nanotubes. Shortening, resulting from long-term sonication, takes place at sites of structural defects present on nanotube surface. It is important to note that the length influences CNT toxicity and cellular uptake [22–23]. Surfactants commonly used for dispersion of CNTs include SDS, CTAB, Triton X-100 or sodium cholate [24–26].

A less known approach is based on the interaction of CNTs with a bis-azo dye – Congo red (CR) [27]. This original procedure was based on the prolonged (2 hours) manual grinding of CR and purified SWNTs in agate mortar with the addition of water to avoid agglomeration of dried CR powder. A greenish black mixture was produced, which was readily dissolved in water [28]. In this paper we present a simpler and more reproducible, sonication-based method of obtaining CR-functionalized SWNTs.

Congo red is best known as an amyloid specific dye, used for years in histochemical analyses for the detection of amyloid fibrils, which – when stained with CR – present characteristic apple-green birefringence under the polarized light microscope. Birefringence implies anisotropy and ordered arrangement of dye molecules bound to regular, beta-structured amyloid fibrils [29–30].

Congo red molecules self-assemble in water solutions producing supramolecular entities stabilized by π–π interactions between aromatic rings [31–33]. Supramolecular Congo red shows unusual protein-binding properties – it preferentially interacts with partly unfolded beta-sheets and thus stabilizes unstable structural states that may emerge as a result of conformational changes linked with the biologic activity of some proteins (e.g., the alpha-1-proteinase inhibitor, antigen-bound antibodies, cell-surface receptors) [29,34–35]. Congo red supramolecular assemblies can incorporate (through intercalation) other molecules – especially those containing planar, aromatic rings – e.g., doxorubicin, rhodamine B, other bis-azo dyes [36–38]. Thus CR could play a role of a carrier that indirectly links these molecules to CNTs, provided that its supramolecular character is preserved upon binding to the surface of the carbon nanotube. Such CR-conjugated CNTs could show increased drug capacity as compared to otherwise solubilized CNTs.

Functionalized single-wall carbon nanotubes (SWNTs) are currently intensely studied as promising drug delivery systems for cancer therapies due to such their properties as: the ability to penetrate cell membrane [16–17], high drug capacity [8–9], selective retention in the tumour [21], reduced toxic effects of the drug [5,20]. The major advantage of carbon nanotubes, as compared to other potential drug carriers, is based on their high drug-binding surface (both inner and outer) and the possibility of chemical modification (functionalization) of the nanotube surface and adjustment of their length [8]. Both functionalization and shortening improve the biocompatibility of nanotubes and increase their retention time in the organism. The retention time of carbon nanotubes is longer than that of PLGA particles or immunoliposomes which are quickly taken up by the reticuloendothelial system cells, thus decreasing the efficiency of drug delivery to the diseased tissue. Lipophilic SWNTs can easily penetrate through cellular membranes. CNTs are characterized by high mechanical strength and heat conductibility and their magnetic and optical properties may be the basis of their simultaneous use as drug carriers and sensitizers in photodynamic therapy.

The aim of our studies is to explain whether CR-functionalized SWNTs could be used as drug delivery systems. Molecular dynamics study of SWNT–CR interactions has already been published [39]. These authors studied several combinations of parameters in order to assess how the SWNT diameter and CR density affect the structure and stability of SWNT–CR conjugates at various pH conditions. The results show that CR binds strongly to the SWNT surface and the SWNT–CR conjugates are thermodynamically stable and that pH changes significantly affect the binding energies of the adsorbed CR. Here we present the experimental analysis of functionalization of nanotubes with Congo red, aimed to explain the role of dye supramolecularity and the mechanism of nanotube–CR interaction.

## Results and Discussion

### Properties of SWNT–CR complexes – the role of CR supramolecularity

**Figure 1:**
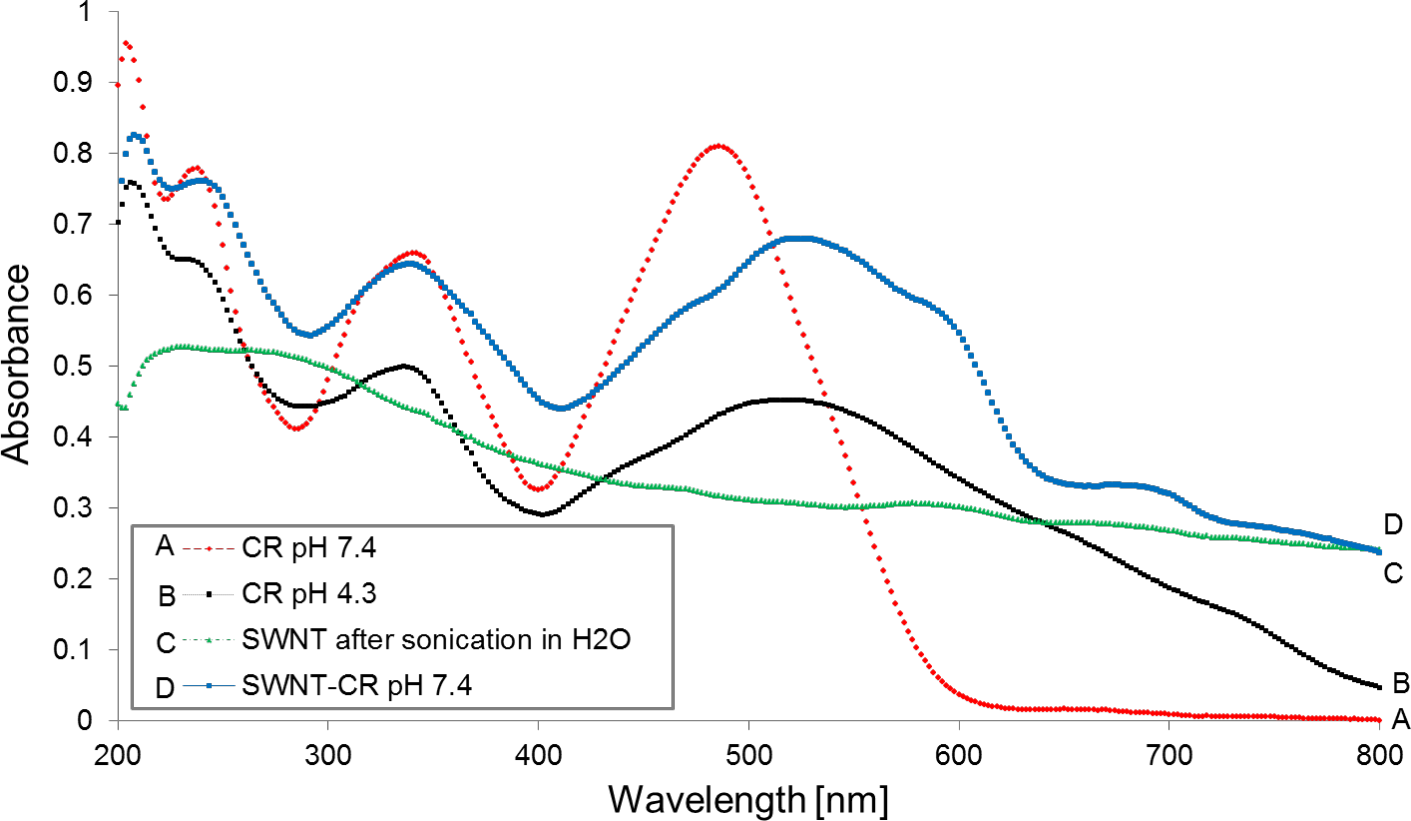
Absorption spectra of free Congo red (A) and SWNT-bound Congo red (D) (0.05 M Tris/HCl buffer, pH 7.4); Congo red spectrum after acidification to pH 4.3 (B); free SWNT suspension spectrum (C).

Comparison of the absorption spectra of free CR and CR bound to SWNTs shows that the binding to the carbon nanotube surface causes a significant change in the Congo red absorption spectrum (Figure 1, curves A and D). This change resembles that caused by acidification (Figure 1, curve B), although pH of the SWNT–CR sample remains unchanged (7.4). The absorption maximum shifts from 490 to 510 nm, leading to the colour change from red to purple.

**Figure 2:**
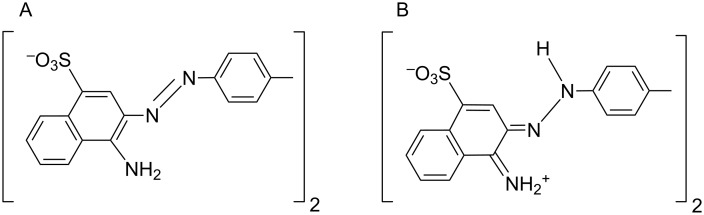
Congo red structure: (A) at physiological pH; (B) ionization of the amino groups at acidic pH and conversion to the quinoid form leading to the change of colour.

In both cases – protonation at decreased pH and adsorption to SWNTs – the shift of the absorption maximum is caused by a change in the electron structure of CR molecule during transition to the quinoid form (Figure 2) which is consistent with the results presented in [40].

The experimentally determined pK value for Congo red amino group pK_NH2_ equals 5.0 [29]. This result suggests the direct interaction of CR aromatic rings with the surface of carbon nanotubes, which are known to be good electrical conductors.

**Figure 3:**
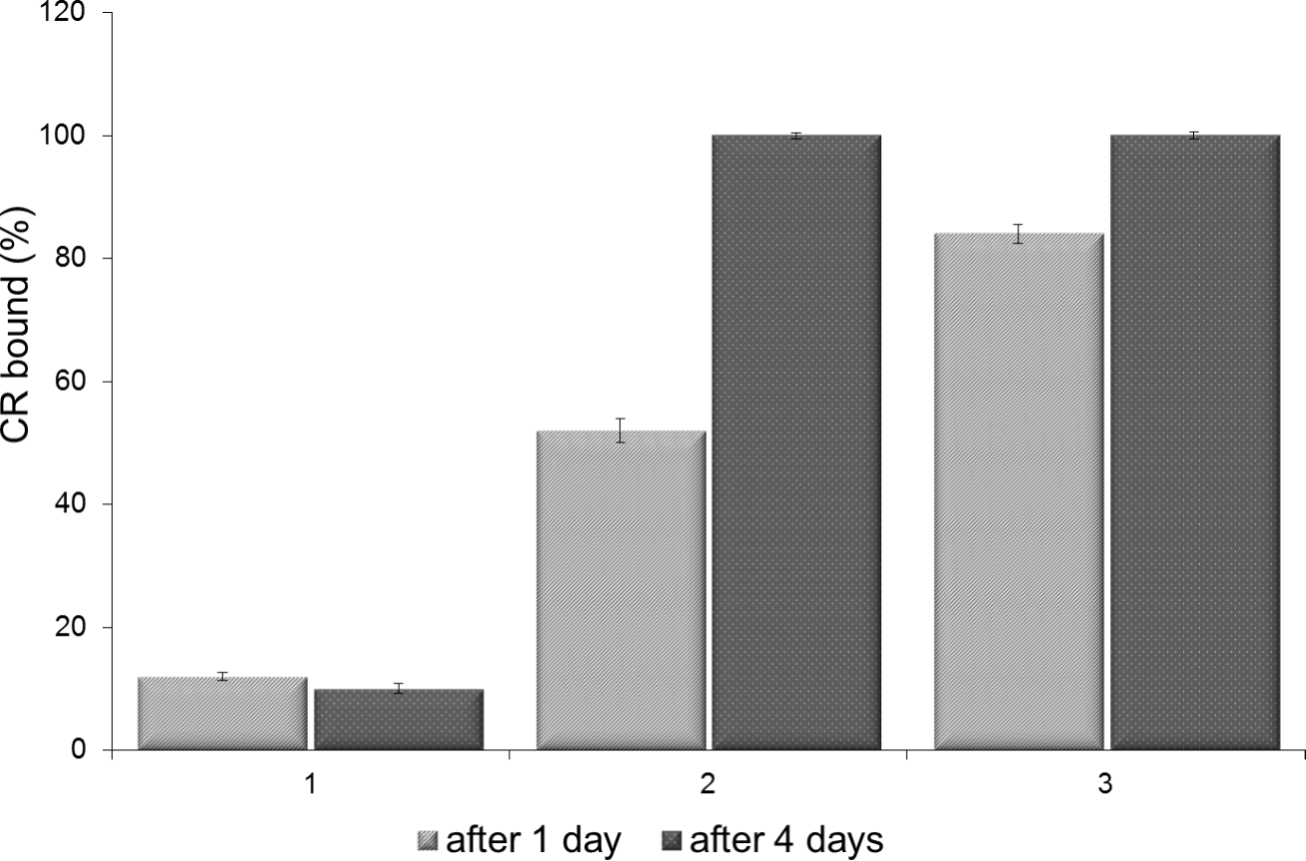
Binding of CR to SWNTs in solutions of different ionic strength. All samples were in a 0.05 M Tris-HCl pH 7.4 buffer with: 1) no NaCl; 2) 0.145 M NaCl; 3) 0.264 M NaCl. The amount of CR added to the nanotube portion was taken as 100%.

Supramolecularity of Congo red in water solutions strongly depends upon the ionic strength of the solvent. At low ionic strength, negative charges of sulphonic groups are not shielded by counterions and the tendency towards formation of the supramolecular structure is low. Addition of salt (e.g., 0.15 M NaCl) stabilizes the interaction between CR molecules and increases its supramolecularity [32]. Figure 3 presents the effect of addition of salt on the amount of CR bound to SWNTs (0.05 M pH 7.4 Tris/HCl buffer with no NaCl and at 0.145 M and 0.264 M NaCl) after 1 or 4 days of incubation at room temperature.

In Figure 3, 100% equals the total amount of CR added to the sample (1 mL of 2 mg/mL CR added to 1 mg of SWNTs, followed by sonication and filtration, as described in the Experimental part).

At low ionic strength (no NaCl) the interaction of CR with SWNTs is low – dispersion of SWNTs is not observed and there are no changes in the absorption spectrum of CR. At both 0.145 M and 0.264 M NaCl the efficiency of CR binding is high, but complexes are formed faster at the higher salt concentration.

### Congo red binding capacity of SWNTs

**Figure 4:**
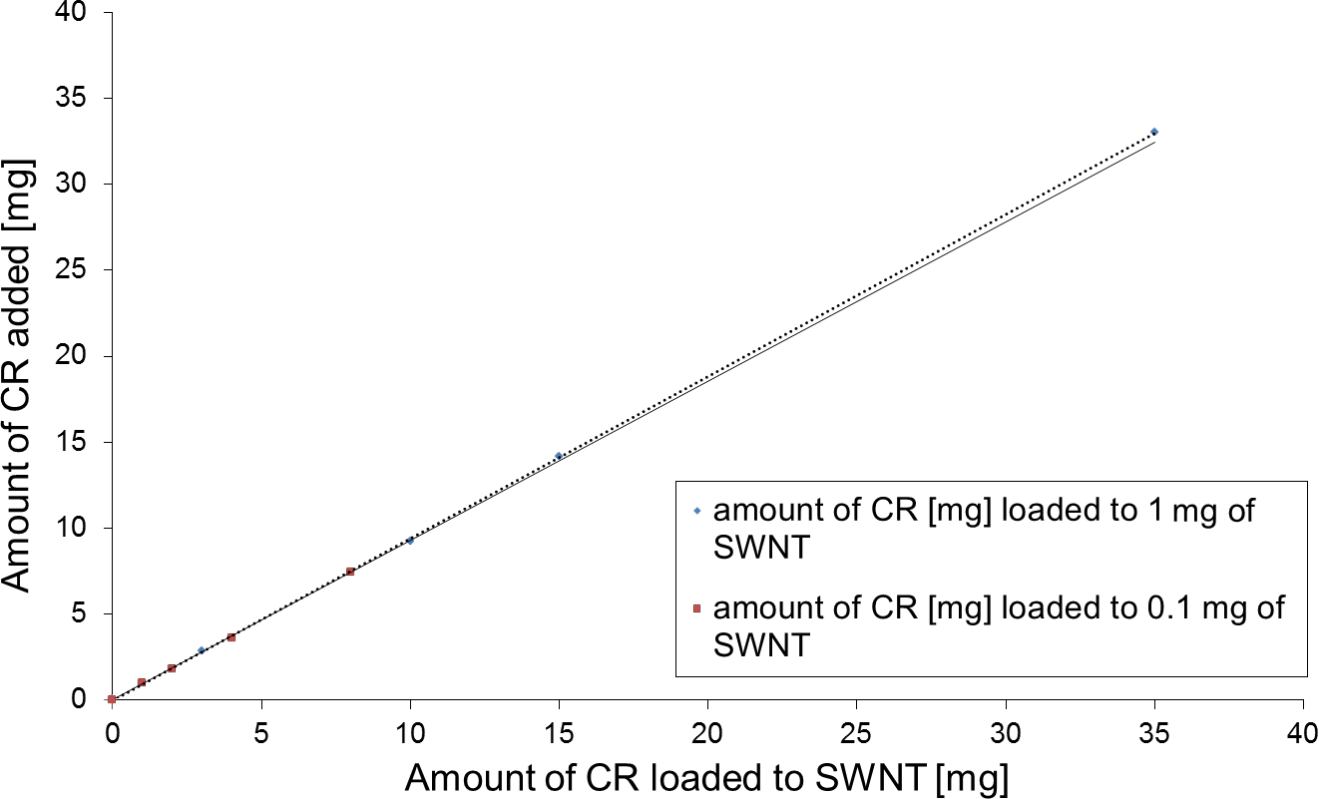
Binding of an increasing amount of CR by a constant amount of SWNTs (0.1 or 1 mg).

Analysis of samples containing SWNT–CR complexes formed in presence of increasing amounts of CR added to the constant amount of SWNTs (0.1 or 1 mg), followed by sonication and filtration as described in the Experimental part, led to the conclusion that the amount of bound CR is proportional to the amount of added CR and that no saturation point can be achieved (Figure 4).

**Figure 5:**
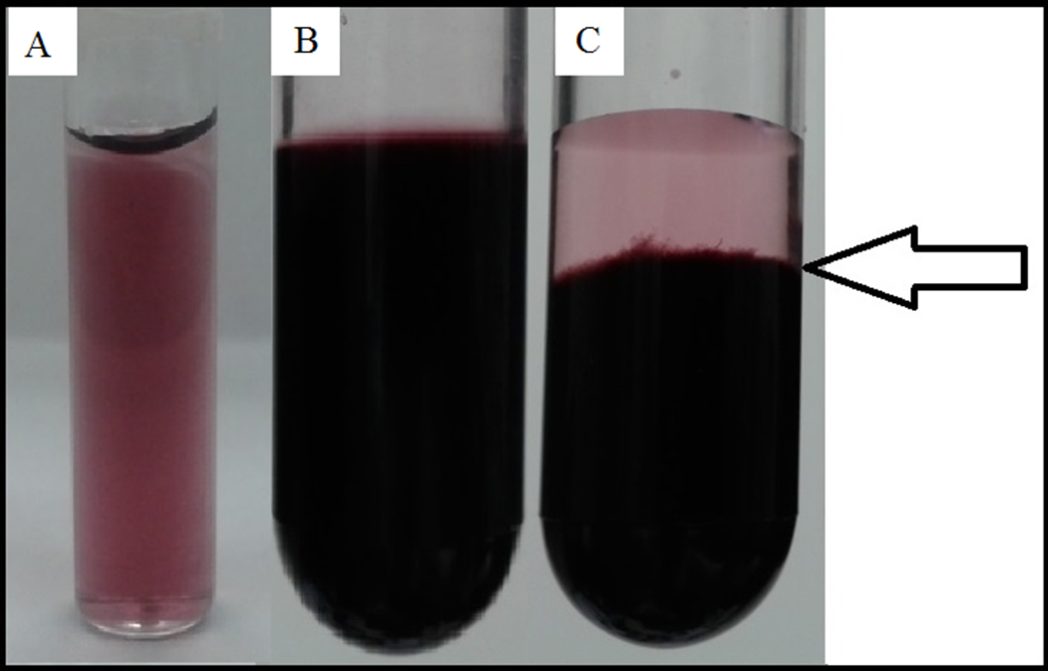
SWNT–CR complexes formed at different CR/SWNT ratios. Macroscopic images of complexes filtered on PTFE membrane and suspended in pH 7.4 0.05 M Tris/HCl buffer. (A) 1 mL of 2 mg/mL CR added to 1 mg of SWNTs – the complex retained its colloidal stability for up to 3 months; (B) 1 mL of 5 mg/mL CR added to 1 mg of SWNTs – the gel-like, time-sedimenting (arrow) (C), but easily dispersed complex was observed.

Surprisingly, 0.1 mg of nanotubes could bind even 30 mg of CR, leading to the formation of a complex that showed a gel-like consistency and sedimented within a couple of days to the lower part of the test tube but could be easily dispersed by gentle mixing. This suggests the formation of a “network” created by interacting SWNTs and supramolecular CR (Figure 5B,C).

### SWNT–CR complex stability – DSC analysis

Differential scanning calorimetry (DSC) analysis of heat flow changes for SWNT–CR complexes was performed in order to evaluate the stability of the complexes formed at different CR/SWNT ratios. The results were recorded with a calorimeter working in power compensation mode.

CR molecules in the supramolecular (liquid-crystalline) form are ordered, but retain partial freedom of movement. Both the translational and rotational freedom of the molecules increases with increasing temperature. Using DSC we can observe changes in the heat capacity that accompany heating or cooling of the sample, and thus analyse the melting or the formation of supramolecular structures for both free CR and CR attached to the carbon nanotube surface.

We analysed heat capacity changes in two cycles of temperature changes: heating after fast cooling and heating after very slow cooling. Free CR (2 mg/mL or 5 mg/mL) and SWNT-bound CR (the same amount) were analysed. All samples were made using 0.05 M Tris/HCl pH 7.4, 0.264 M NaCl buffer.

The thermograms were analysed with respect to the formation of the ordered structures - which lowers the heat capacity (downward peaks), and melting of supramolecular structures (endothermic processes – upward peaks).

**Figure 6:**
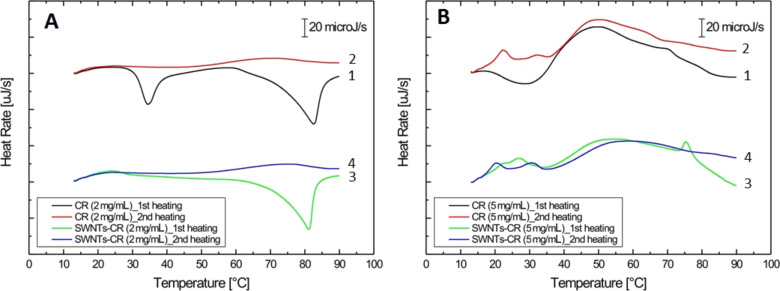
Calorimetric profiles obtained for free CR and CR bound to SWNTs: (A) 2 mg/mL CR; (B) 5 mg/mL CR. The first heating was always preceded by rapid cooling; second heating was preceded by controlled, slow cooling (1 °C/min).

Thermograms for free and SWNT-bound CR at two concentrations (2 and 5 mg/mL) are presented in Figure 6.

The “first heating” thermogram was registered for the sample which was previously quickly cooled while the “second heating” thermogram concerns the sample which was previously cooled slowly, in a controlled way – 1 °C/min. The “first heating” curve for free CR sample (Figure 6A, curve 1) shows a phase transition at 30–40 °C (maximum at 34.5 °C). This peak is much wider (shows a higher value of full width at half maximum) for the higher CR concentration (Figure 6B, curve 1). In both cases the phase transition reflects the formation of organized, liquid-crystalline structures from less ordered assemblies present in a fast-cooled solution. The rate of cooling is of big importance in case of supramolecular compounds. Abruptly cooled solutions of molecules that show a high self-assembling capability – like CR – create rather disordered assemblies which may be converted to an ordered and more stable (optimum) form as the temperature is raised slowly.

The presence of a wider peak in the thermogram for the sample with higher CR concentration suggests the existence of less homogenous structures. This transition is not observed for the “second heating” samples (Figure 6A and B, curves 2), as the controlled, slow cooling (1 °C/min) favours the formation of highly ordered, and relatively stable, ribbon-like assemblies of CR molecules.

The small upward peak at 22.3 °C observed during the second heating for the 5 mg/mL CR solution (Figure 6B, curve 2) can reflect the breakdown of an undefined, low stability supramolecular structure of CR. Melting of an ordered supramolecular CR takes place at about 50 °C – this is clearly visible for the 5 mg/mL sample (Figure 6B, curve 1 and 3). In previously published results (for 10 mg/mL CR solutions) melting took place at 40 to 60 °C, depending upon the ionic strength of the solution [32].

When thermograms for free CR and SWNT-bound CR are compared (Figure 6) the following differences are observed: (1) The lack of 34.5 °C transition in the SWNT–CR complex – probably caused by the interaction of ordered CR at the nanotube surface (Figure 6A). This interaction takes place when the sonicated sample is left in room temperature. High enthalpy of the registered exothermic transition suggests that nanotubes may be responsible for the ordering of bound CR; (2) CR added to SWNTs at a lower (2 mg/mL) concentration creates complexes with nanotubes, which become more ordered and stable when the sample is cooled after the first heating. This results in the absence of the phase transition at 80 °C – Figure 6A, curve 4; (3) At a higher CR concentration (5 mg/mL), the nanotube-bound CR is also more stable than free CR. The presence of a wide peak at 50–60 °C caused by breakdown of the supramolecular structure is observed. It probably corresponds to CR molecules being attached indirectly to the nanotube, through face-to-face interaction with CR adhering to the nanotube surface (Figure 6B).

### Thermal stability of SWNT–CR complexes

**Figure 7:**
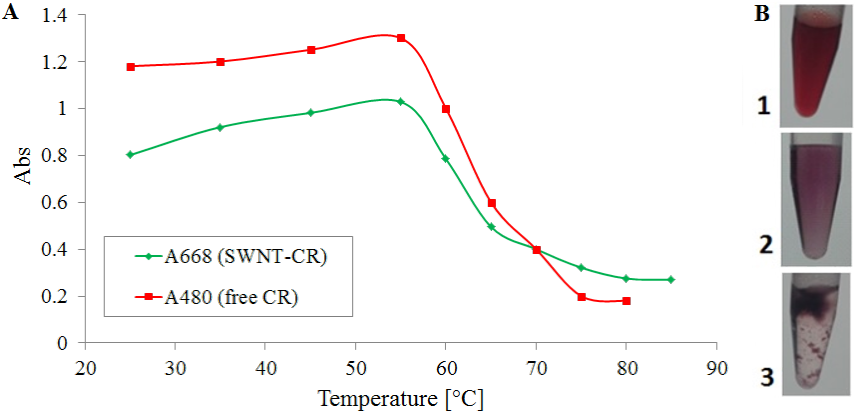
Precipitation of the SWNT–CR complex upon incubation at increasing temperature; (A) absorption spectra of the supernatant – decrease of absorption at 480 nm means that CR is not released from the complex; (B) macroscopic images of the samples: 1. CR solution; 2. SWNT–CR complex at room temperature; 3. SWNT–CR complex after heating above 60 °C.

SWNT–CR complexes (1 mg SWNTs + 1 mL of 2 mg/mL CR, sonicated and filtered) are stable at temperatures up to 55 °C. Above that temperature the precipitation of nanotubes is observed (Figure 7B.3), but CR is not released into the solution). Figure 7A shows the decrease in absorbance of the supernatant for both free CR (at 480 nm) and for the SWNT–CR complex (at 668 nm) after 20 minutes incubation at a given temperature.

The temperature at which the precipitation effect is observed correlates with the melting temperature for the CR supramolecular form [32]. The reason of aggregation of nanotubes is unclear, but most probably the release of CR molecules from SWNT–CR complexes at increased temperature allows the aggregation of nanotubes, followed by the reassociation of CR with the surface of SWNT bundles (thus the supernatant is colourless).

### Dynamic light scattering analyses

Dynamic light scattering (DLS) applied to SWNT–CR complexes analysis does not provide precise measurements of the nanotube diameter, but allows to compare SWNT samples dispersed using different factors. We used nanotubes dispersed with sodium cholate as control and a SWNT–CR complex sample obtained through the exchange of cholate to CR. The CR was added to nanotubes initially dispersed with sodium cholate, the sample was dialyzed leading to the formation of the SWNT–CR complex as the cholate was removed. This procedure ensures the same dispersion of the control sample and the SWNT–CR complexes. Both SWNTs and SWNT–CR probes were 10 times diluted before measurements.

**Figure 8:**
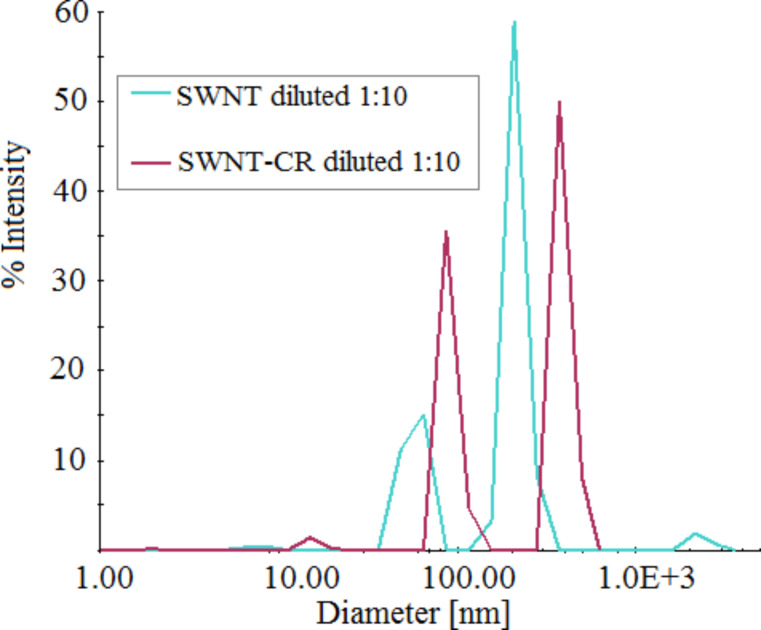
Dynamic light scattering (DLS) measurements: comparison of SWNT–CR complexes and sodium cholate dispersed SWNTs (control).

As shown in Figure 8, both cholate-dispersed SWNTs and SWNT–CR complexes present several maxima. The SWNT–CR peaks are shifted towards higher values, as the interaction with supramolecular CR increases the nanotube diameter.

### Microscopic analyses

SWNT–CR complexes were studied using various microscopic techniques. All analyses showed that interaction with CR significantly increases the diameter of the nanotube, but in an uneven way, which suggests the presence of large, supramolecular CR assemblies attached to the nanotube surface.

**Figure 9:**
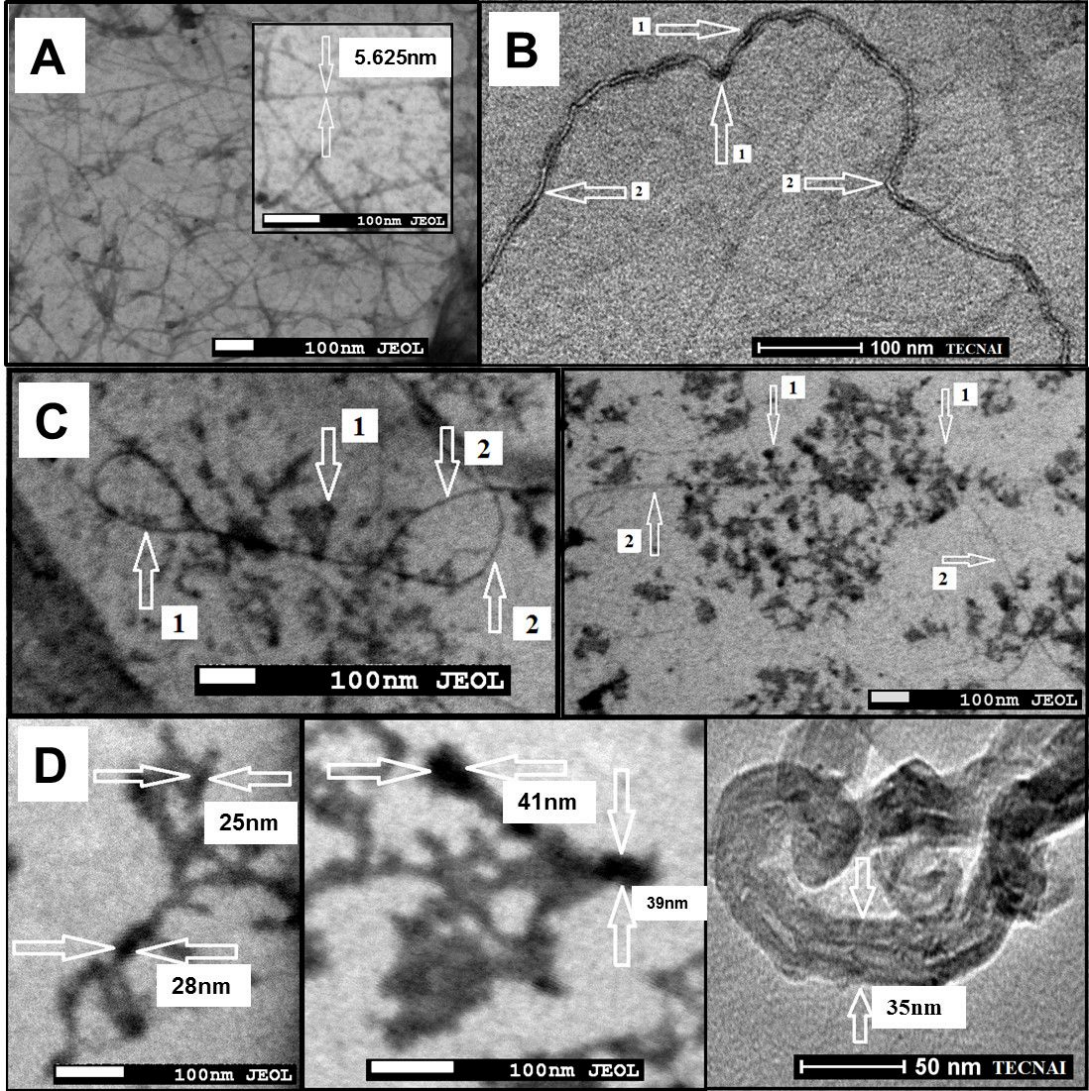
Transmission electron microscope images. (A) SWNTs dispersed by sodium cholate; the diameter of the individual SWNT – 5.6 nm; (B) SWNT–CR complex created at low 1:1 CR/SWNT ratio (1 mg SWNTs + 1 mL of 1 mg/mL CR). Arrows indicate (1) CR binding sites and (2) CR-free areas (Tecnai, Osiris); (C) SWNT–CR complexes formed at 5:1 CR/SWNT ratio (1 mg SWNT + 1 mL of 5 mg/mL CR): nanotubes unevenly covered with CR are visible. Arrows indicate (1) CR binding sites and (2) CR-free areas. The diameter of SWNT–CR complex exceeds 20 nm in thickest areas (Jeol); (D) SWNT–CR complexes formed at very high 10:1 CR/SWNT ratio (1 mg SWNTs + 1 mL of 10 mg/mL CR): CR covers the entire surface of carbon nanotubes, the diameter of SWNT–CR complex exceeds 40 nm in thickest areas.

SWNTs dispersed with sodium cholate (control) have approximately 5 nm in diameter as observed with the transmission electron microscope (Figure 9A).

Congo red-covered nanotubes formed at a 1:1 CR/SWNT ratio (in this case all added CR was adsorbed to the nanotubes) are well dispersed, although their surface is not entirely covered with the dye. CR-covered areas show increased diameter – 9 to 17 nm (Figure 9B). Complexes formed at higher, 5:1, CR/SWNT ratio (all added CR was also adsorbed to the nanotubes) show uneven distribution of adsorbed CR, suggesting that areas that have already bound CR molecules can preferably bind subsequent molecules of the supramolecular dye. Thus the nanotube diameter can locally increase up to 25 nm, while other areas remain bare (Figure 9C). At a CR/SWNT ratio of 10:1 nanotubes are fully covered with CR and their diameter increases to 28 nm but locally may reach about 40 nm (Figure 9D).

**Figure 10:**
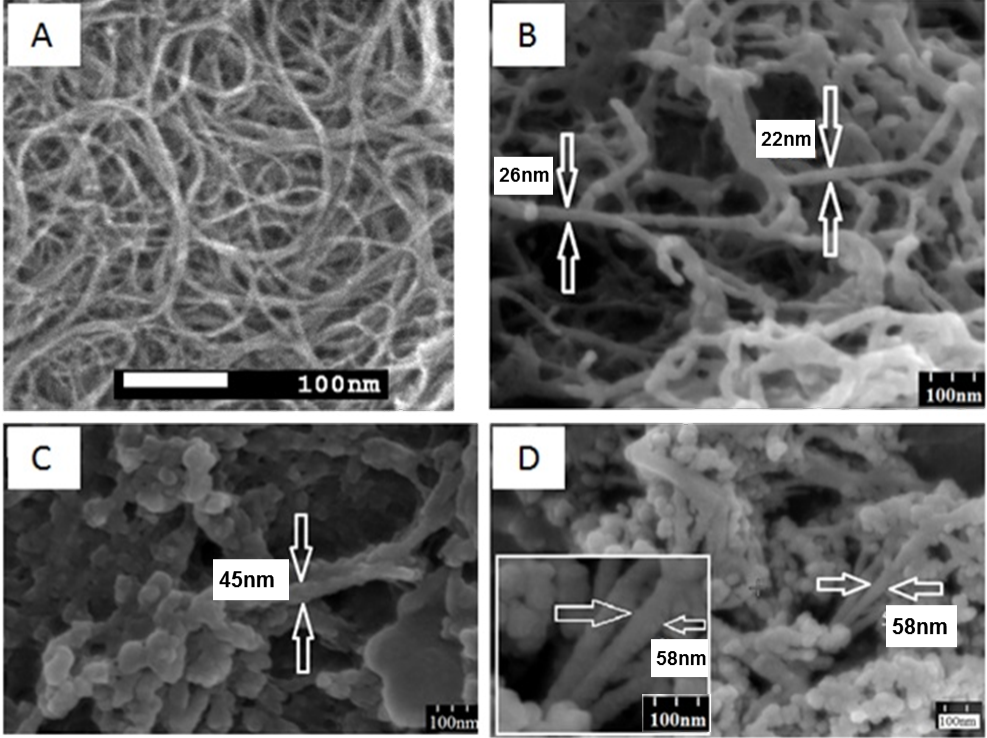
Scanning electron microscope images. (A) undispersed, raw SWNTs; (B) SWNT–CR complexes formed at high 5:1 CR/SWNT ratio (1 mg SWNTs + 1 mL of 5 mg/mL CR): CR covers the entire surface of carbon nanotubes*,* the diameter of the SWNT–CR complex exceeds 20 nm; (C, D) SWNT–CR complexes formed at very high 10:1 CR/SWNT ratio (1 mg SWNTs + 1 mL of 10 mg/mL CR): CR covers the entire surface of carbon nanotubes*,* the diameter of the SWNT–CR complex exceeds 40 nm and more.

Uneven loading of SWNTs (Figure 10A) with CR is also seen in scanning electron microscope images. CR-covered areas present bulges in Figure 10B–D. The diameter increases up to 50 nm, which is in accordance with TEM results.

**Figure 11:**
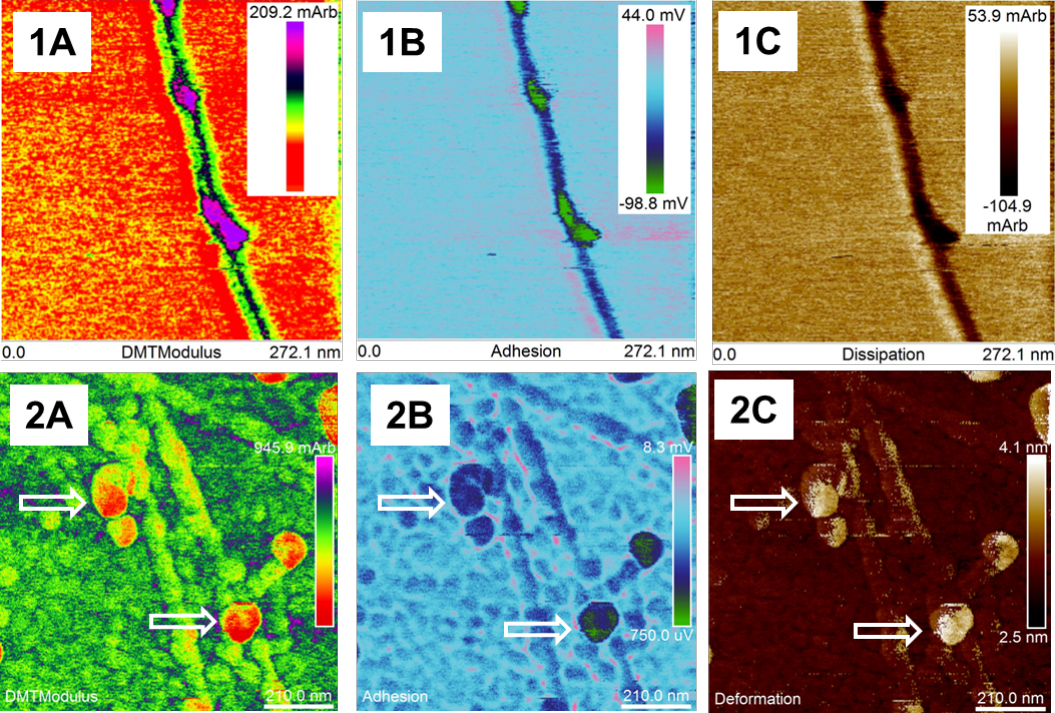
AFM analyses of sodium cholate dispersed SWNTs (1) and SWNT–CR complexes (2); maps of mechanical parameters: (1A; 2A) elasticity (Young modulus), red – the lowest value; (1B; 2B) adhesion (green – lowest value); (1C) dissipation (dark – the lowest value); (2C) deformation (white – the highest value). Arrows point to local thickening and decreased stiffness (high plasticity) on the surface of the CR-covered nanotubes.

Transmission and scanning electron microscopy images are consistent with AFM results. Using the AFM technique we compared cholate-dispersed (control) and CR-dispersed SWNTs (obtained at 5:1 CR/SWNT ratio) analysing mechanical parameters such as elasticity, adhesion, dispersion (dissipation energy) and deformation (Figure 11).

Cholate dispersed SWNTs form a monolayer at the mica surface (Figure 11.1) while SWNT–CR complexes form a thick layer with single nanotubes protruding above its surface (Figure 11.2). This can be explained by gel-like properties of the complex formed at high CR/SWNT ratio (see Figure 5B,C), which is a network of nanotubes and supramolecular CR.

Elasticity measured as the stiffness range is specified in arbitrary relative units. Elasticity reached of about 200 mArb on the free SWNT (black) compared to mica layer (red) (Figure 11.1A). In Figure 11.2A we observed a thick layer of SWNTs covered by CR with elasticity of about 450 mArb, but also bulbs which show much lower stiffness and higher plasticity. These bulbs were characterized by smaller adhesion energy (Figure 11.2B) and highest deformation (Figure 11.2C) and in consequence higher plasticity compared to free SWNTs.

**Figure 12:**
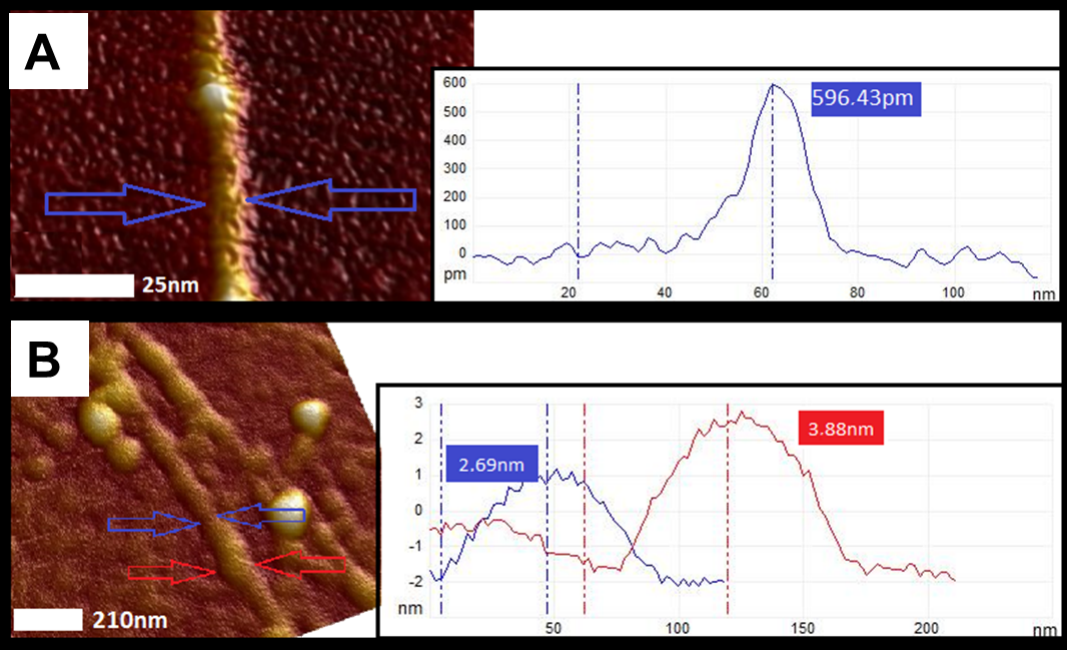
Diameter of nanotubes as measurements of height (AFM cross section analysis): (A) the diameter of a single (cholate dispersed) nanotube is about 0.6 nm; (B) the diameter of CR covered SWNTs ranges from 2.7 to approx. 4 nm at the sites of weak load.

The diameter of cholate dispersed nanotubes performed by AFM was 0.6–1 nm, while for CR-dispersed SWNTs 3–4 nm at the sites of weak load (Figure 12). This value may be undervalued due to the thick layer of SWNT–CR, from which protrude measured SWNT–CR. But generally in comparison with the free SWNTs we see an increase in diameter.

## Conclusion

Direct functionalization of carbon nanotubes based on their interaction with Congo red allows for their dispersion in aqueous media and creates complexes in which the supramolecular character of CR is retained. Congo red presents unique properties as it forms ribbon-like supramolecular assemblies which are quite stable in aqueous solutions but simultaneously show some plasticity [35], important not only for binding to carbon nanotubes but also for interaction with proteins (as it can adapt to the protein binding site) [31,34].

At solutions of low ionic strength, when CR supramolecular properties are much weaker, its interaction with SWNTs is greatly decreased. At higher ionic strength of the solution, which favours formation of supramolecular CR assemblies, dispersion of SWNT with CR is observed and the number of CR molecules bound to the nanotubes correlates with ionic strength of the solution, confirming the role of supramolecularity (Figure 3).

Two possible mechanisms of supramolecular SWNT–CR interaction were considered: (1) „edge-to-face” – in which the edge of supramolecular ribbon-like CR assembly interacts with the nanotube surface and (2) „face-to-face” – in which aromatic rings of individual CR molecules adhere to the SWNT surface. These directly adsorbed CR molecules can interact with subsequent CR molecules producing carbon nanotubes decorated with protruding ribbon-like structures of supramolecular CR. Both experimental data and theoretical analyses [39] support the “face-to-face” model.

Stacking of dye’s aromatic rings to the carbon lattice of the nanotube produces a significant shift of the absorption spectrum (and the red to purple colour change). This shift, similar to that observed at acidic pH, is observed although pH 7.4 is retained. This could be explained by the flow of electrons from the nanotube to CR molecules - both to those directly adsorbed (trough π–π interactions between CR and a carbon nanotube) and to the ones indirectly bound as elements of the supramolecular assembly. Since carbon nanotubes are conductors, they can serve as donors of electrons to CR, which switches to the quinoid form (Figure 2) and changes colour.

Molecular modelling analysis [39] showed how aromatic rings of CR molecules can adhere to the nanotube surface.

The flow of electrons from the nanotube to CR can locally change the properties of the nanotube surface, making its interaction with other CR molecules less probable. This may explain why TEM images of SWNT–CR complexes formed at 5:1 CR/SWNT ratio show both bare regions and areas covered with a thick CR sheath (Figure 9C). The diameter of CR-loaded SWNTs increases with increasing CR/SWNT ratio – as observed in TEM, SEM, AFM and DLS – supporting the face-to-face mode of the interaction between CR and the SWNT surface.

Differential scanning calorimetry analyses show that the interaction of CR with nanotubes stabilizes the supramolecular structure of CR. As the sonicated SWNT/CR mixture is left at room temperature and the CR binds to the nanotube surface, CR molecules become ordered (and the 35 °C transition is not observed in DSC analysis – Figure 6 A). The ordering of CR is also responsible for the birefringence of the SWNT–CR complexes observed using polarized light microscopy (data not shown). CR molecules bound indirectly (and thus more loosely) to the nanotube as distant elements of the CR ribbon, which are more abundant at higher CR/SWNT ratio, can dissociate from the SWNT–CR complex as the temperature is raised, producing the transition peak at about 55 °C (Figure 6B).

Unexpectedly, we could not estimate the maximum amount of CR that could bind to the nanotube (Figure 4) as even at 350:1 (CR/SWNT, w/w) most of the added CR was trapped in the complex. Interestingly, at high CR concentrations the complex shows gel-like properties, which can be explained through formation of a network containing nanotubes connected by supramolecular CR.

Supramolecular ribbon-like assemblies created by CR show a capability to bind (intercalate) many different compounds, provided that at least a part of a molecule is a planar aromatic ring (e.g., Rhodamin B, Titan yellow) [36–38]. Since many drugs show such a structural feature, CR-nanotube complexes can be considered as possible drug-carriers. The ability of nanotubes to bind high amounts of Congo red would be advantageous, as it could increase the drug-binding capacity of the nanotube carrier.

## Experimental

### Materials

Single-wall carbon nanotubes (SWNTs, purity >90%, length 700 nm, diameter 0.7–0.9 nm), Congo red (CR, 96% purity) and sodium cholate were obtained from Sigma. Polytetrafluoroethylene (PTFE) membranes (0.2 µm pore size) were purchased from MERCK Millipore. All other reagents used were of analytical grade.

### Dispersion of SWNTs by non-covalent attachment of supramolecular Congo red

1 mL of aqueous CR solution (2 or 5 mg/mL) was heated for 3 min at 100 °C, slowly cooled to room temperature and added to 1 mg of SWNTs. The mixture was sonicated for 2 h (in cooled water bath) and then left for 24 h at room temperature. The SWNT–CR complex was separated from the unbound CR by filtration on PTFE membrane. This allowed to obtain well-dispersed CR-coated SWNTs that were directly used for further experiments. SWNT–CR complexes obtained in the method described above retained their colloidal stability for at least 3 months (stored at 4 °C or at room temperature).

The amount of CR bound by SWNTs was determined spectrophotometrically based on the amount of CR in the eluate as compared to the total amount of CR added to the sample. UV–vis absorption spectra were recorded on a Cary 300Bio Varian spectrophotometer. Absorption coefficient for CR: ε_489_ = 50.46 M^−1^·cm^−1^. In order to avoid errors caused by clogging of the membrane (in case of samples containing higher amounts of CR) the filtering procedure was repeated twice with a fresh filter. For some experiments sodium cholate was used in order to create the control sample – carbon nanotubes dispersed without CR.

### Characterization

#### Differential scanning calorimetry (DSC)

SWNT–CR complexes for the DSC analysis were prepared as follows: 1 mg of SWNTs was suspended in 1 mL of 2 or 5 mg/mL CR solution (in 0.05 M Tris/HCl buffer pH 7.4, 0.264 M NaCl), sonicated and filtered as described above to obtain carbon nanotubes differing in the degree of loading with CR. As the controls we analysed thermal capacities of Congo red supramolecular ribbon-like structures formed in 2 mg/mL and 5 mg/mL solutions. The analysis was performed using NANO DSC Series III System (model 6300) with Platinum Capillary Cell (TA Instruments) equipped with Nano DSCRun software. In order to avoid the formation of air bubbles during the heating, the samples were degassed for 10–15 min before the measurement using the negative pressure of 0.3–0.5 atm. A reference chamber was filled with a buffer used for the analysed sample. All the measurements were performed at 3 bar pressure in a 0.33 mL capillary cell. Data was collected in the range of 10–90 °C at 1 °C·min^−1^ scan rate for both heating and cooling. In order to ensure the thermodynamic equilibrium, the measuring system was equilibrated for 10 min at an initial temperature (10 °C in the heating mode, and 90 °C in the cooling mode). Baselines were made by recording scans for reference samples (appropriate buffers). Each set of data was analysed using NanoAnalyze software supplied by TA Instruments.

#### Dynamic light scattering (DLS)

The interaction of CR with carbon nanotubes was studied using the DynaPro NanoStar (Wyatt Technology). The measurements were done after 3 min incubation at 25 °C within the instrument. Each measurement – 20 acquisitions lasting 5 seconds each – was performed in triplicate.

#### Transmission electron microscopy (TEM), scanning electron microscopy (SEM), atomic force microscopy (AFM)

Scanning and transmission electron microscopy was conducted on a Jeol JSM-7500F with EDS INCA Pentaflex 3 adapter to analyse the elemental composition of a sample. Scanning electron microscopy was performed on Hitachi S-4700 model equipped with a system of elemental composition analysis based on X-ray energy dispersion (Noran Instruments). High-resolution transmission electron microscopy (HR-TEM) was conducted on a FEI Tecnai Osiris G2 20 TWIN. Atomic force microscopy (AFM) was conducted on a Dimension FastScan or Dimension ICON (Bruker Nano, Santa Barbara, California). Surface of carbon nanotubes was visualised in Peak Force QNM (Quantitative NanoMechanics) mode, allowing the simultaneous mapping of the topography and mechanical properties.

The samples were dialyzed for 24 h to deionized water, then a 1 µL droplet of SWNT–CR suspension was placed onto a freshly cleaved mica surface (for AFM measurements) or onto a 300 mesh copper TEM grid (for TEM measurements) and dried overnight in a desiccator. The samples for SEM measurements were dried and sprayed with gold in a technical vacuum sputter.

AFM measurements were performed using SNL-10 probe (type A microcantilever) with silicon tip mounted on a silicon nitride microcantilever. The elasticity constant of the microcantilever was 0.32 N/m. The nominal radius of the tip before the measurement was 2 nm. PeakForce QNM (PF QNM) performs a very fast curve at every pixel in the image. Using PF QNM mode visualization, maps of both the surface and mechanical properties were generated simultaneously. This allowed to obtain following characteristic: stiffness (elasticity) of the material (DMT Modulus; the higher value of the modified Young's modulus means the more stiff material), adhesion forces (it allows mapping the maximum force with which the microcantilever is attracted by the sample at the time of retract of the piezoelectric scanner), deformation (the indentation of the surface under the tip), dispersion (dissipation, loss of energy of microcantilever of scanning probe when passing through different areas on the sample surface).
